# Advances in Knowledge and Management of Immune-Related Adverse Events in Cancer Immunotherapy

**DOI:** 10.3389/fendo.2022.779915

**Published:** 2022-03-22

**Authors:** T. Anders Olsen, Tony Zibo Zhuang, Sarah Caulfield, Dylan J. Martini, Jacqueline T. Brown, Bradley C. Carthon, Omer Kucuk, Wayne Harris, Mehmet Asim Bilen, Bassel Nazha

**Affiliations:** ^1^ Winship Cancer Institute, Emory University, Atlanta, GA, United States; ^2^ Department of Hematology and Medical Oncology, Emory University School of Medicine, Atlanta, GA, United States; ^3^ School of Medicine, Emory University, Atlanta, GA, United States; ^4^ Division of General Internal Medicine, Department of Medicine, Massachusetts General Hospital, Harvard Medical School, Boston, MA, United States

**Keywords:** immunotherapy, irAE, oncology, pharmacology, immune checkpoint inhibitors, immunotherapy combined therapy, steroids

## Abstract

Immune-oncologic (IO) therapy has revolutionized the treatment and management of oncologic disease. Immunotherapy functions by enhancing the host immune-systems ability to endogenously clear malignant cells, however, this activation can also lead to immune-mediated damage to healthy native tissues. These side effects are known as immune-related adverse events or irAEs and can even present with phenotypes similar to autoimmune diseases. IrAEs are the major consequence of checkpoint inhibitors and can have a significant impact on a patient’s cancer treatment and long-term quality of life. The management of these irAEs follows a similar approach to autoimmune diseases. More specifically, the management is akin to that of autoimmune disease exacerbations. While there is an array of immune-suppressing agents that can be used, steroids, immunomodulators and IO discontinuation are cornerstones of irAE management. The exact approach and dosing are based on the severity and subtype of irAE presented. Within recent years, there has been a push to better prevent and manage irAEs when they arise. There has been an additional effort to increase the number of steroid-sparing agents available for irAE treatment given the consequences of long-term steroid therapy as well as patient contraindications to steroids. The goals of this review are to summarize irAE management, highlight significant advances made in recent years and emphasize the future directions that will optimize the use of IO therapy in oncology.

## Introduction

**Figure 1:**
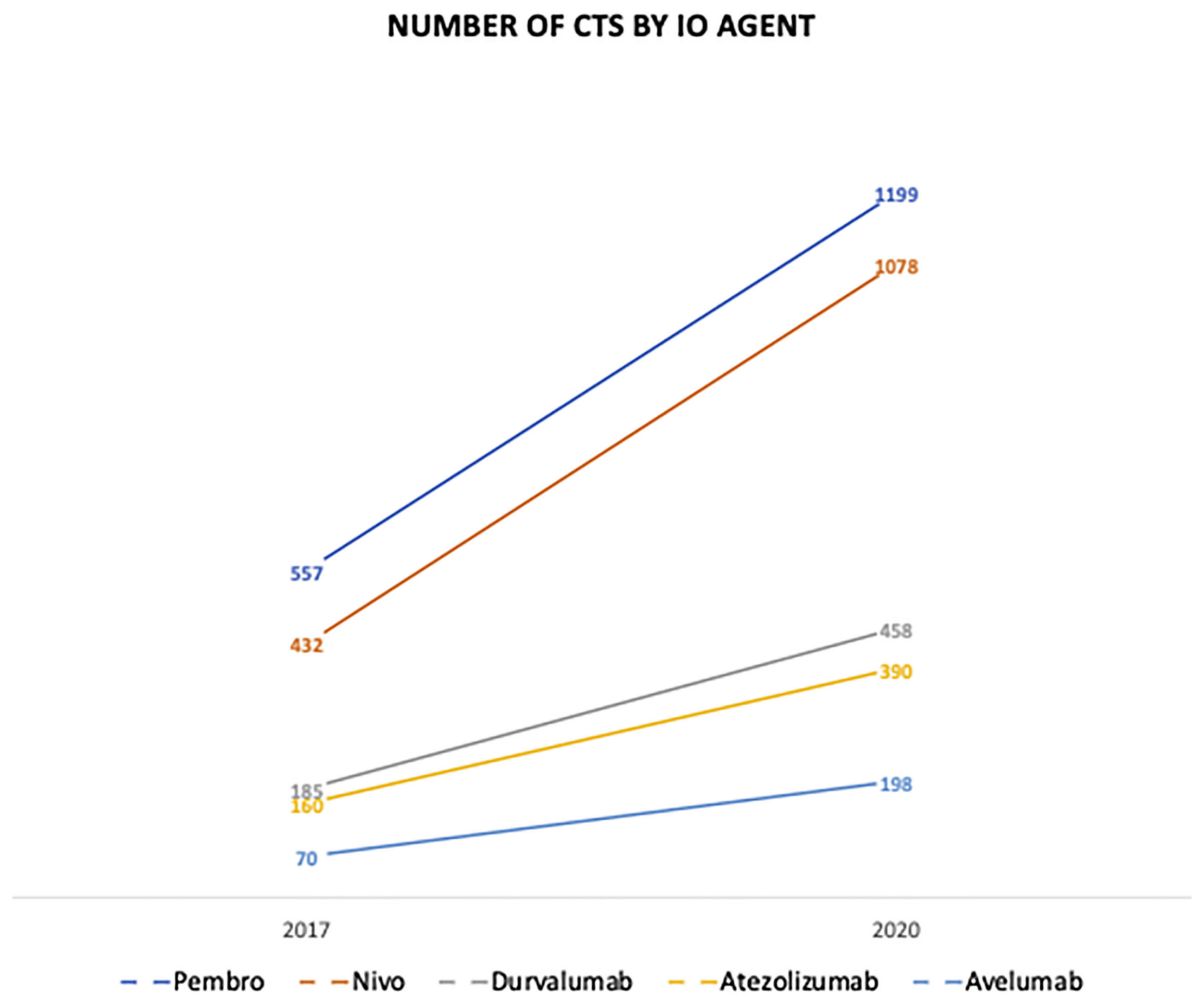
The number of clinical trials in IO by ICI agent from 2017-2020. IO, Immuno-Oncologic Therapy; ICI, Immune-Checkpoint Inhibitor.

Immunotherapy is often seen today as a relatively novel agent in the realm of cancer care. However, immune-acting anti-cancer agents have been used since the late 1970s with BCG in treating bladder cancer as a form of local immune-oncologic (IO) therapy (1). Since then, the immunotherapy arsenal saw an explosion of new agents which includes small molecules, targeted monoclonal antibodies (MCAs), reproduced cytokines and, more recently, oncolytic viruses and CAR-Ts (1). These new class of agents, especially immune checkpoint inhibitors (ICIs), has shown promise in the management of advanced or metastatic cancers. **Figure  1** (2) visually displays the significant growth in the number of clinical trials for ICI agents from 2017 to 2020 (2). The IO drug category success lies in its ability to provide durable responses in subsets of treated patients. However, for as long as IO therapy has been available for treatment, side effects associated with immune over-activation have followed suit. Given the numerous and recent FDA approvals for ICIs, irAEs associated with these specific agents are particularly relevant in the contemporary management of malignancies. **Table 1** (3) includes a list of FDA approved IO approved agents by malignancy. Tumor cells often can evade natural immune-mediated surveillance and cell death by presenting antigens that cause T cell senescence and exhaustion (4). ICIs act through three major cellular pathways in the tumor micro-environment by inhibiting these interactions at the programmed death-1 (PD-1) or PD-ligand-1 (PD-L1) or cytotoxic T lymphocyte antigen-4 (CTLA-4) interfaces (5). The most prominent of these treatments include the following FDA-approved MCA based therapies ipilimumab (anti-CTLA-4), nivolumab (anti-PD-1), pembrolizumab (anti-PD-1), cemiplimab (anti-PD-1), atezolizumab (anti-PD-L1) and avelumab (anti-PD-L1), among others.

**Table 1:** Examples of US FDA Approved ICI Agents and Indications by Cancer Type.

US FDA Approved Immune-Checkpoint Inhibitors	ICI or IO Combination Regimens
**Cancer Type**	
Squamous Cell Head & Neck Cancer	2L Nivolumab or Pembrolizumab after chemo
Cutaneous Squamous Cell Carcinoma	1L Pembrolizumab with platinum chemo
	1L Cemiplimab
Malignant Melanoma	Adjuvant ipilimumab, nivolumab or pembrolizumab
	1L Ipilimumab, nivolumab or pembrolizumab
	1L Combo: Nivolumab + ipilimumab
Non-Small Cell Lung Cancer (NSCLC)	Unresectable stage III: durvalumab after chemo-radiation
	1L Pembrolizumab for TPS > 50%
	1L non-squamous NSCLC Combo:
	Pembrolizumab + pemtrexed & platinum
	Atezolizumab + bevacizumab, paclitaxel & carboplatin
	1L squamous NSCLC Combo:
	Pemborlizumab + carboplatin & paclitaxel
	2L Pembrolizumab TPS > 1%
	2L Atezolizumab or nivolumab
Advanced Microsatellite Instability-high cancer (MSI-H/dMMMR)	2L Nivolumab in CRC
	2L Nivolumab + ipilimumab in CRC
	2L Pembrolizumab in any MSI-H/dMMR cancer
Small Cell Lunger Cancer (SCLC)	1L Atezolizumab with chemo
	2L Nivolumab after platinum chemo
Advanced Renal Cell Carcinoma	1L Combo: Nivolumab + ipilimumab Avelumab + axitinib Pembrolizumab + axitinib 2L Nivolumab after anti-angiogenic agent (ex: VEGFi)
Locally Advanced/Metastatic Urothelial Cancer	1L Pembrolizumab, atezolizumab
	1L/2L: Pembrolizumab, atezolizumab, avelumab, dervalumab, nivolumab after platinum salt
Hepatocecllular Carcinoma	2L Nivolumab or pembrolizuamb after sorafenib Combo: Atezolizumab + bevacizumab
Triple Negative Brest Cancer	1L Atezolizumab + paclitaxel protein-bound PD-L1 > 1%
Gastric & Gastric-Esophageal Junction (GEJ) Cancer	2L dMMR or MSI-H: gastric and GEJ
	2L Pembrolizumab CPS > 10; esophageal and GEJ
	3L Pembrolizumab CPS>1; gastric
Classical Hodkin Lymphoma	2L Pembrolizumab post-transplant (Auto or Allo-graft)
Cervical Cancer	2L Pembrolizumab CPS > 1
Endometrial Cancer	Pembrolizumab + lenvatinib (second-line)
Merkel Cell Carcinoma	2L Avelumab or pembrolizumab
Primary Mediastinal B-Cell Lymphoma	3L Pembrolizumab

IO, Immuno-Oncologic Therapy; ICI, Immune Checkpoint Inhibitors.

IrAEs can occur at any organ system with a wide range of presentations (6). In a meta-analysis studying IO side effects, irAEs were split into three basic categories: general (fatigue, diarrhea, and rash), organ specific (colitis, hepatitis, pneumonitis, myocarditis, etc.) and musculoskeletal (arthritis, arthralgia, joint pain, etc.) (7). While general irAEs tended to be the most common, this study emphasized the clinical importance of organ-specific irAEs due to their higher rates of associated mortality (cardiac and hepatitis specifically) and unclear associations with therapeutic efficacy (example: vitiligo or skin and endocrine irAEs were associated with clinical benefit while hepatic, pulmonary and gastrointestinal irAEs were not) (8). All in all, ICIs offer a dynamic and effective treatment option for advanced oncologic disease. However, the oncologic benefits that these agents provide must be weighed against potential irAEs and their subsequent treatments.

## Epidemiology

**Figure 2:**
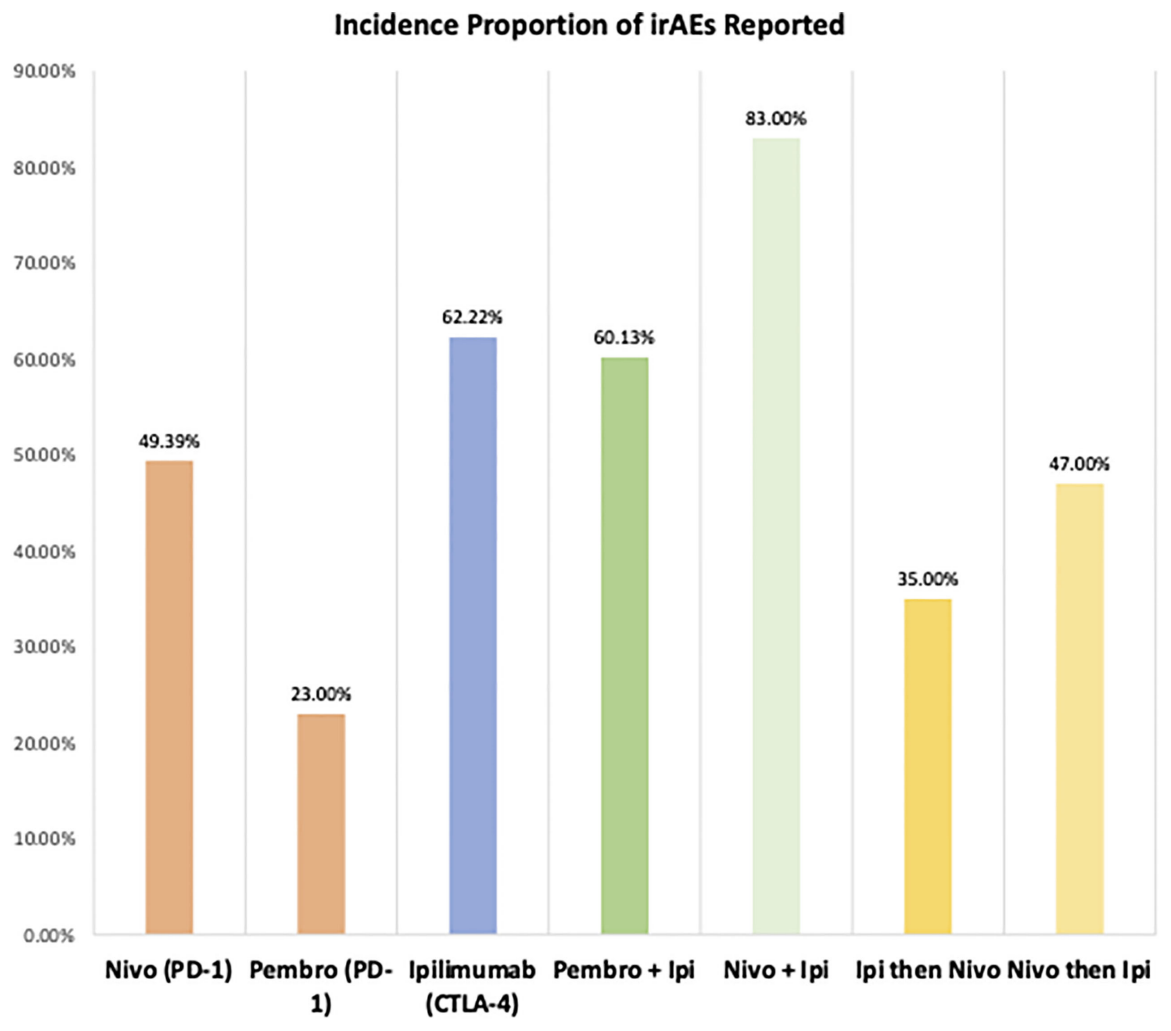
Proportion Incidence of All IrAEs by Mono/Dual-IO Agents Cohorts Using Data from Advanced Melanoma IO Trials. Proportion of all irAEs (Grade 3 or less) incidence amongst 9 major advanced melanoma RCTs using ICIs organized by mono/dual therapy studied. IO, Immuno-Oncologic Therapy; RCT, Randomized Control Trial; irAE, Immune-related adverse event; ICI, Immune-checkpoint Inhibitor; Pembro, pembrolizumab; Nivo, nivolumab; Ipi, ipilimumab; PD-1, Programmed Death Receptor-1; PD-L1, Programmed Death Receptor Ligand-1.

The percentage of patients who are eligible for ICI therapy has increased dramatically from 1.54% of cancer patients in 2011 to 43.63% in 2018 (9). Within major phase II and phase III clinical trials for ICIs, the combined incidence of adverse events ranged from 54 to 76% depending on the specific ICI agent studied (10). With the approval of many ICI combination regimens, this incidence is only expected to compound upon previous rates (11). Based on pooled data studies, irAEs tend to occur within 2-16 weeks (median time of onset) after the initiation of ICI therapy (12). However, some reports have found irAEs within days or even years of treatment initiation. With this recent explosion of ICIs, the incidence of irAEs has had a similarly rapid rise with a recorded 13,000 irAE cases in 2018 (13). Of these, 60% were associated with the three following agents: iplimumab, nivolumab and pembrolizumab (13). **Figure 2** (2, 14) displays the percent incidence of irAEs amongst trial populations based on ICI class (dual and monotherapies) from data collected through 9 melanoma clinical trials (14).

### Broad Management Principles

The management of these irAEs, as stated earlier, follows similar principles to the treatments used for acute autoimmune pathologies. Corticosteroids and IO treatment discontinuation are the most frequently deployed tactics when it comes to managing irAEs (15). In addition, several immunomodulating agents, used more frequently in rheumatology, can be useful for specific and severe irAEs. In patients experiencing irAEs associated with endocrine organs, management often necessitates direct hormone replacement (depending on the severity of the iatrogenic endocrinopathies). However, the continuation of ICI is possible if the anti-cancer effect is sustained (16).

### irAE Proposed Mechanisms

The potential mechanisms through which irAEs occur is related to the function of the ICIs themselves as well as the general activation of immunogenic T cells. **Table 2** (17) includes irAEs that are more strongly associated with certain IO mono and dual-therapy regimens. While the exact pathways are still unclear, the CTLA-4 and PD-1/PD-L1 ICI classes both appear to suppress the function of regulatory T cells (Tregs), which are specialized helper T cells that suppress the cellular immune system (18). These inputs can lead to increased immuno-activation and decreased cellular surveillance over autoreactive interactions within the humoral and cellular-based arms of the immune system (19). Uniquely, the CTLA-4 agents specifically increase the 17 T helper cell counts, a specialized T cell that secretes the proinflammatory cytokine interleukin-17 (18, 19). This laboratory findings is also seen in CTLA-4 knockout mice These factors taken with the increased release of pro-inflammatory cytokines have been associated with decreased selection against auto-reactive T cells and antibody producing B cells. Additionally, cross-reactive antigens on healthy tissues can also occur through a similar mechanism to *molecular mimicry* with the source of specious antigens coming from malignant cells (20, 21).

**Table 2:** Immune-related Adverse Events (irAEs) Associations by Class of Immune Checkpoint Inhibitor (ICI) or ICI-Combination Regimens.

US FDA Approved Immune-Checkpoint Inhibitors	ICI Agents	Associated irAEs
Agent Target		
**Monotherapy**		
CTLA-4	Ipilimumab	Generally greater incidence of irAEs and of higher severity compared to PD-1/PD-L1. Most common relative to PD-1/PD-L1: **GI-associated (diarrhea/colitis), hypophysitis fatigue, opthalmologic and dermatologic**
PD-1	Cemiplimab	Most common relative to CTLA-4: **Rheumatic, auto-immune (re-/intial-activation), musculoskeletal, thyroid, pulmonary, infusion-related reactions, oral mucositis and myasthenia gravis**
	Nivolumab	
	Pembrolizumab	
PD-L1	Atezolizumab	
	Avelumab	
	Durvalumab	
Comparable between PD-1/PD-L1s and CTLA-4s		**Transaminitis/hepatic, pancreatic, neurologic**
**Combination regimens**		
Dual-IO (irAEs significantly elevated; generally most irAEs are elevated)		**GI-associated (diarrhea/colitis), hepatic, endocrine (thyroid), fatigue, nausea, rash,**
IO and VEGF (antiangiogenic agent)	IO + Cabozantinib (MCA), ramucirumab (MCA) axitinib (TKI), pazopanib (TKI), cabozanitnib (TKI)	**Negative cardiac effects (HTN, thrombophilia, bleeding), transaminitis/hepatic and GI-associated**
IO and Chemo	Platinum-based, microtubule-targeting agents	**Lower overall risk of Aes grade 3 or higher relative to chemo alone; except for: neuropathy,**
IO and EGFR	Erlotinib, afatinib, loratinib	**Pulmonary and hepatic**

IO, Immuno-Oncologic Therapy; ICI, Immune Checkpoint Inhibitors; irAE, Immune-related adverse events; CTLA-4, Cytotoxic T-Lymphocyte Antigen-4; PD-1, Programmed Death Receptor-1; PD-L1, Programmed Death Receptor Ligand-1; VEGF, Vascular Endothelial Growth Factor; Chemo, Chemotherapy; GI, Gastrointestinal.

Bolded terms represent most common irAEs associated with the given agents.

Interestingly, some studies have found a correlation between certain irAEs and improved oncologic outcomes (8). This suggests that certain irAEs can act as positive prognostic indicators for the degree of immune-activation instigated by ICIs.

When considering the impact of combination strategies, the use of multiple IO therapies appears to further intensify the degree of immune function leading to enhanced T cell penetrance into malignant tissue whilst also increasing the rate and severity of irAEs (22).

## irAE Screening and Diagnosis

The widespread use of ICIs required treating clinicians to have a depth of knowledge into oncology, the immune system, and the complex intersect between the two. Due in part to the intricate array of organ-specific irAEs, a multi-disciplinary approach is often required to provide the best possible care for patients receiving IO therapies. Prior to treatment initiation, screening candidates for immunopathology is crucial. A detailed personal and family history including medication review can help elucidate any past auto-immune or rheumatologic diseases (23). Even if symptoms have never been displayed, patients with an elevated risk of autoimmunity are hypothesized to benefit from an autoantibody panel although this practice is not common. While these pre-treatment screenings can help identify patients at risk for irAE, these assays do not fully contraindicate these patients from receiving ICIs given the potential benefit to oncologic outcomes (24). This has been a specific area of advancement in the ICI clinical space that emphasizes old and novel potential biomarkers discovered through pharmacovigilance and multi-omic signaling data (25).

Diagnosis of irAEs is also a crucial aspect to ICI therapy, with subtle and low-grade irAE being commonly missed. Due to the wide range of presentations, high morbidity and potential mortality of certain irAEs, clinical suspicion for a potential adverse event after ICI-initiation should be promptly pursued. This evaluation could require comprehensive laboratory panels, specialty-specific consultations as well as radiologic (CT/MRI) and tissue-specific testing/biopsies (26). Clinicians should be particularly keen for symptoms linked to organ-specific irAEs occurring in cardiac, pulmonary and liver tissues (18, 27, 28). Although rare, these reactions are associated with high mortality compared to other irAEs.

## irAE Grading and Organ-Specific Management

Within IO clinical trials and in clinical practice, the severity of irAEs are graded using the Common Terminology Criteria for Adverse Events (CTCAE) developed by the US National Cancer Institute (NCI). IrAEs are measured from grade 1-5 correlating to mild, moderate, severe, life-threatening, and deadly, respectively (29). The approach to treating irAEs is dependent upon the specific tissue affected by the irAE and the severity grade based on the CTCAE classification system. Generally speaking, grade 1 events can be managed with temporary (rarely permanent) cessation of treatment or treatment continuation with close monitoring. Grade 2 events can also be managed with treatment discontinuation; however, they could require specialist referrals and steroids. Grades 3-4 should have therapy halted and receive a glucocorticoid taper in most situations. Patients with grade 4 reactions should be considered for inpatient/intensive care unit (ICU) admission for additional support and monitoring. Endocrine irAEs are the exception to this and are primarily treated with hormone-replacement. Second and third-line immune-modulating agents can also be used depending on the type of irAE experienced. Summaries of the therapies for each irAE can be seen in **Table 3** (26, 30) with dosing and special indications noted.

**Table 3:** IrAE Management Options by Organs Affected.

US FDA Approved Immune-Checkpoint Inhibitors		Therapy Options (Grade-specfic)	Dosing or Diagnostics
IrAE (bold) or Side Effect (unbolded)	Line of treatment	*denotes steroid resistant irAE management	if specified
Cardiac	1st	high-dose IV corticosteroids + oral taper	1g/day + 4-6 week PO taper
	2nd	abetacept*	
		mycophenolate*	
		IVIG*	
		alemtezumab*	
		infliximab*	
		anti-thymocyte globulin*	
Pulmonary	1st	Oral steroids	1-2 mg/kg/day PO
	2nd	infliximab (G3-4)	5mg/kg IV (repeated 2 wks later)
		IVIG (G3-4)	
		mycophenolate mofetil (G3-4)	1-1.5 g BID PO
Dermatologic (rash)	1st	topical emollients (G1)	
		antihistamines (G1)	
		lidocaine patches (G1)	
		medium potency topical steroids (G1)	
		oral steroids	0.5 mg/kg/day
		high potency topical steroids	0.5-1 mg/kg/day
	2nd	Arepitant	
		Omalizumab	
Pruritis		Gabapentinoids (G2+)	
Bullous Dermatitis (BD)	1st	high potency topical steroids (G1)	0.5-1 mg/kg/day
		oral steroids (G2)	1-2 mg/kg/day
SJS/TEN/BD G3-4	1st	oral steroids (G3-4)	1 g/kg/day for 3-4 days
	2nd	IVIG*	
Gastrointestinal (colitis/diarrhea)	1st	Loperamide (G1)	
		Diphenoxylate (G1)	
		Atropine (G1)	
		Mesalamine (G1)	
		Cholestyramine (G1)	
		Oral steroids (G2)	1-2 mg/kg/day
		IV steroids (G3-4)	1-2 mg/kg/day
	2nd	infliximab* (G2-4)	If no response to steroids after 2 weeks
		vedolizumab* (G2-4)	
Hepatic	1st	Oral steroids	1-2 mg/kg/day
	2nd	Mycophenolate (G2-4)	
Pancreas	1st	Oral steroids (G3)	0.5-1 mg/kg/day
		Oral steroids (G4)	1-2 mg/kg/day
Renal	1st	Oral steroids (G2)	0.5-1 mg/kg/day
		Oral steroids (G2-4)	1-2 mg/kg/day (used in G2 if persistent)
	2nd	Azathioprine*	
		Cyclophosphamide*	
		Cyclosporin*	
		Infliximab*	
		Mycophenolate*	
Central Nervous System	1st	Oral steroids (mild)	0.5-1 mg/kg/day
		Oral steroids (severe)	2 mg/kg/day
Positive LP for oligioclonal bands		IVIG/Plasmapharesis	
Positive LP for autoimmune encephalitis antibody		Rituximab	
Peripheral Nervous System	1st	Oral steroids (G2)	0.5-1 mg/kg/day
		Oral steroids (G2-4	2-4 mg/kg/day (used in G2 if progressive)
Neuropathic Pain	1st	Gabapentinoids	
		Duloxetine	
Ophthalmologic	1st	Local steroid drops	
		Oral steroids (severe)	
Endocrine: do not require steroids in 1st line			
Thyroid			
Hyperhyroid	1st	Beta blocker	10-20 mg q4-6h
Hypothyroid	1st	Levothyroxine	If TSH > 10
Hypophysitis	1st	Appropriate hormone replacement therapy	
Musculoskeletal (arthralgia, arthritis, etc.)	1st	NSAIDs	
		Oral steroids (mild)	10-20 mg daily for 2-4 weeks
		Oral steroids (severe)	0.5-1 mg/kg/day for 2-3 weeks
	Adjunct to steroid per rheumatology	Infliximab	
		Methotrexate	
		Tocilizumab	
		Sulfasalazine	
		Azathioprine	
		Adalimumab	
		Etanercept	
		Hydroxychloroquine	
Pain		Non-opioid pain regimen	1-2 mg/kg/day
Myalgias/Myositis	1st	Oral steroids	
	2nd	IVIG*	
		Plasmapharesis*	
		Infliximab*	
		Rituximab*	
		Mycophenolate*	
Pain		Non-opioid pain regimen	

IO, Immuno-Oncologic Therapy; ICI, Immune Checkpoint Inhibitors; irAE, Immune-related adverse events; IVIG, Intravenous immunoglobulin.

### Dose Specific Guidelines by irAE Type

In this next section, we review the most recent treatment algorithms for the different irAEs using expert consensus guidelines on ICI toxicity management and highlight the advances in each. However, prior to examining the details of management, it is important caveats regarding irAE classification and diagnosis. The grading system is far more useful to organ-specific irAEs and less useful for the ambiguous general (fatigue) and subclinical endocrine (thyroid, adrenal) irAEs. Additionally, there lacks a consensus for the documentation and clear classification for irAEs, as many studies have found poor agreement between providers on how to best classify irAE timing, type, and severity (31).

### Cardiac irAEs

Cardiac reactions require immediate cardiology (preferably cardio-oncology) consultation, ECG, telemetry, echocardiograms as well as biomarkers for cardiac damage (troponin, CK-MB, BNP) and inflammation (ESR, CRP) (26, 32). New guidelines also recommend viral titers especially for COVID-19. If myocarditis is suspected or can be confirmed with biopsy, subsequent work-up includes ICI discontinuation, high-dose IV corticosteroids for 3-5 days 1g/day (methylprednisolone) and subsequent oral steroids for 4-6 weeks with surveillance of previously collected laboratory markers (32). ICU admission and temporary or permanent cardiac pacing may also be required at any grade. Major advancements for cardiac irAEs include the inclusion of immunomodulators if pathology is unresponsive to steroids in the first 24 hours. There have also been updates that divide myocarditis from pericarditis/pericardial effusion, the latter of which is managed with the standard of care for pericardial inflammation once myocarditis is excluded (32). Newer agents for steroid-refractory cases include abatacept, mycophenolate, IVIG, alemtuzumab, infliximab and anti-thymocyte globulin (32).

### Pulmonary

Pulmonary irAE or pneumonitis management is divided between mild (grade 1-2) and moderate-severe (grades 3-4) pneumonitis. Grade 1 reactions require IO hold, reassessment in 1-2 weeks (history/physical, pulse oximetry) and a possible chest CT with follow-up imaging in 4-6 weeks if symptoms do not resolve (32). Grade 2-4 reactions must have an initial minimally invasive work-up which includes a thorough infection rule-out (nasal swab for respiratory viruses, sputum culture, acid-fast stain, blood culture, urine antigen testing for *pneumococcus and legionella)* and possible chest CT imaging with repeat imaging in 3-4 weeks (32, 33). If symptoms worsen or do not improve with IO hold, invasive evaluation can be considered, which includes bronchoscopy with biopsy or bronchoalveolar lavage and broad-spectrum antibiotics for empiric infection coverage (26, 32). Prednisone/methylprednisolone 1-2 mg/kg/day is recommended every 3-7 days with pulse oximetry and clinical monitoring (26, 32). If no improvement is observed within 2-3 days of treatment for grade 2, patients are treated as having a grade 3 reaction (26, 32). Grades 3-4 require permanent IO discontinuation, inpatient care; an urgent pulmonary and infectious disease consultation; and the same minimally-invasive/invasive protocol (26, 32). Oral corticosteroids (methylprednisolone 1-2 mg/kg/day) should be given and tapered over 6 weeks or more (26, 32). Major advancements in pulmonary irAE management include the division of minimally invasive and invasive evaluations in diagnosis, as well as the inclusion of steroid-sparing agents if steroids provide no improvement within 2 days for grades 3-4 pneumonitis (26, 32, 33). These include infliximab 5 mg/kg IV (dose can be repeated in 2 weeks), IVIG or mycophenolate mofetil 1-1.5 g BID with taper.

### Dermatologic irAEs

Milder skin reactions include maculopapular rashes and pruritis, which are relatively common reactions. Their management is dependent upon the severity of the reaction.

For maculopapular rashes grades 1-2, ICI treatment can be continued, and patients can be provided topical emollients, antihistamines/lidocaine patches for pruritis and moderate-high potency topical steroids on affected areas (26, 32). If unresponsive to topical steroids, patients can be given oral prednisone 0.5 mg/kg/day (32). For grades 2, immunotherapy treatments are held, and patients are treated with high-potency topical steroids or oral prednisone 0.5-1 mg/kg/day (26, 32). Gabapentinoids can be given alternatively for grade 2 pruritis. Grade 3-4 reactions require urgent dermatology consultation, high-potency topical steroids and inpatient care for grade 4 reactions (26, 32). Grade 3 and higher may also consider MCA therapies (aprepitant, omalizumab) if symptoms are refractory to first-line agents (26, 32). Major advancements include the use of gabapentinoids in the management of irAE-associated pruritis and rash.

More worrisome dermatologic reactions include blistering or bullous dermatitis (BD) and Stevens-Johnson syndrome (SJS) or toxic epidermal necrolysis (TEN) (32).

For BD, SJS and TEN an urgent dermatologic consultation is strongly recommended and, if unavailable, a skin biopsy should be obtained (26, 32). For BD, grade 1 reactions are managed with high potency steroids to affected areas and ICI cessation (26, 32). Grade 2 reactions can also be given oral steroids 0.5-1 mg/kg/day (prednisone/methylprednisolone) or rituximab if no improvement is observed after 3 days (32). For grades 3-4 BD or signs of SJS/TEN, inpatient care is required along with urgent consultation with dermatology, ophthalmology, and urology (32). Additionally, patients should be given oral steroids 1-2mg/kg/day (prednisone/methylprednisolone) or IVIG 1/g/kg/day divided in doses over 3-4 days if refractory to steroids (26, 32). The management of dermatologic irAEs has not faced significant changes in recent years outside of the addition of gabapentinoids as a non-steroid alternative treatment option for managing pruritis or rashes.

### Gastrointestinal irAEs

Gastrointestinal (GI) irAEs can be divided into two major categories. Ones that involve the intestines (colitis/diarrhea) and those that involve major digestive organs such as the liver and the pancreas. For all grades, a thorough GI infection panel is required, which includes nucleic-acid amplification tests (NAATs), bacterial cultures; Ova/parasites for *Giardia, Cryptosporidium* and *E. Histolytica*; and a *C. difficile* toxin (26, 32). For grades 2-4, CT imaging studies and GI consultation should also be considered (32). Major advancements have included the emphasis of adequate hydration, the use of lactoferrin/calprotectin in diagnosis (+/- endoscopy), infection screenings (HIV, hepatitis panel), IV steroids for grade 3-4 for proper absorption and newer steroid-sparing MCAs in moderate-severe irAEs (32).

#### Colitis/Diarrhea

Diarrhea is the most common irAE associated with ICI and is even more common with combination IO regimens (32). For grade 1, immunotherapy is held, adequate hydration is recommended, and patients can be given loperamide or diphenoxylate/atropine for 2-3 days (26, 32). If no improvement is observed, providers should check lactoferrin/calprotectin levels. If lactoferrin/calprotectin levels are positive, patients can be treated as having grade 2 diarrhea/colitis and receive invasive imaging endoscopy (26, 32). If negative, patients are considered grade 1 and can be given mesalamine and cholestyramine in addition to the previous regimen (32). For grade 2 reactions, a GI consultation can be considered (for imaging), and patients are given oral steroids 1-2 mg/kg/day instead of GI motility agents. If no response is observed in 2-3 days, providers can consider adding infliximab or vedolizumab within 2 weeks of steroid initiation (26, 32). For grades 3-4, patients are advised to have an urgent GI consultation, receive inpatient care and given IV methylprednisolone 1-2 mg/kg/day immediately (26, 32). Patients should not be given oral agents due to risks of poor absorption (32). If no response is observed in 1-2 days, steroids should be continued with the addition of infliximab or vedolizumab (32).

For post-toxicity resolution, grade 3 reactions require discontinuation of CTLA-4 agents and consideration of anti-PD-1/PD-L1 classes for future IO treatments (26, 32). Grade 4 reactions lead to permanent discontinuation (26, 32).

#### Liver

Liver damage from irAEs is often associated transaminitis noticed on laboratory screenings with symptomatic presentations being a sign of more severe damage (32). Grading is based off the degree of liver function-test (LFTs) and bilirubin abnormalities relative to the upper limit of normal (ULN). Grade 1 reactions can have IO therapy held and later reassessed (32). The management of reactions that are greater than grade 1, LFTs <3 times the ULN, are split based on the degree of bilirubin elevation (32). Initial management includes exclusion of viral hepatitis re-activation, a screening for a history of Gilbert syndrome, a thorough medication reconciliation of hepatotoxic agents such as acetaminophen, a review of supplements/dietary toxins (alcohol, ashwaghanda and other health supplements), hepatologist consultation and possible abdominal imaging (32). Lower bilirubin levels (1-2 times ULN) can have IO held and oral steroids 1-2mg/kg/day initiated. LFTs are monitored every 2-3 days (32). If no resolution occurs with steroids, mycophenolate can be added. For higher bilirubin levels (3-4 times ULN; more frequently in grade 3-4 reactions) the management is the same as lower bilirubin elevations, except inpatient care and urgent hepatology consultation is recommended as well as permanent IO discontinuation (32). Grade 4 reactions can consider having liver biopsy performed if not contraindicated (32). Infliximab is contraindicated for the treatment of irAE-associated hepatitis according to the NCCN guidelines. Despite lacking specific justification for this recommendation, it is likely due to infliximab’s potential for liver damage which could exacerbate liver function in the setting of an irAE (32). However, this concern has been questioned by smaller studies that have shown some moderate success with infliximab in reducing the severity of liver irAEs (34). Advancements include the use of mycophenolate and MCA contraindications for advanced irAE (26, 32).

#### Pancreas

For irAEs associated with the pancreas the general management is split between asymptomatic amylase/lipase elevations and symptomatic pancreatitis, which is further sub-divided by grades 2-4 (32). For asymptomatic patients, mild elevations (< or = 3 times ULN amylase/lipase) can have IO therapy continued with further evaluation for pancreatitis, which includes an abdominal CT with contrast and/or MRCP, if CT is non-diagnostic. (26, 32) For moderate and severe asymptomatic elevations (moderate: >3-5 x ULN; severe > 5 x ULN amylase/lipase), IO can still be continued after thorough clinical and imaging studies to evaluate for active pancreatitis (32). If no pancreatitis is observed, patient can continue clinical observation with follow-up laboratory and imaging assessments. If pancreatitis is noted clinically or on imaging the degree of inflammation is graded from 2-4. With grade 2 reactions, IO is likely held, and it is recommended that the patient receive IV hydration and GI consultation (32). Grade 3 reactions have a similar management to grade 2, except for a definitive IO hold and oral steroids 0.5-1mg/kg/day (32). Grade 4 reactions require permanent IO discontinuation and higher dose oral prednisone/methylprednisolone of 1-2mg/kg/day (32). The major advancement was the improved classification of asymptomatic patients found to have amylase/lipase elevations rather than any adjustments to overall management itself.

### Renal

IrAEs associated with the kidney are graded based on the degree of creatinine (Cr) elevation. For all grades, it is recommended that patients receive a thorough history for previously nephrotoxic agents (IV contrast, fluid status, UTIs and medications) and that nephrotoxic medications are discontinued, or dose reduced. A spot urine protein/creatinine ratio (Up/Cr) IS also recommended. Grade 1 reactions have Cr elevations 1.5-2 times above baseline or have a > 0.3 mg/dL increase (32). For grade 1, IO is usually held and a follow up Up/Cr is obtained every 3-7 days (32). If no improvement is observed over 2 weeks, nephrology consultation is advised. For grade 2 reactions (Cr 2-3 times baseline), IO is held, nephrology is consulted for thorough glomerulonephritis work-up, Up/Cr is obtained every 3-7 days and renal biopsy is considered prior to starting oral steroids, which are dosed at 0.5-1 mg/kg/day (32). For persistent grade 2 reactions, prednisone/methylprednisolone can be given at 1-2 mg/kg/day (32). For grade 3-4 reactions (>3 times baseline or a > 4 mg/dL increase) a similar approach is taken, except that inpatient care and possible dialysis are indicated, and steroids are initially dosed at 1-2 mg/kg/day (prednisone/methylprednisolone) for 4-6 weeks (32). If no resolution is observed after this treatment period, azathioprine, cyclophosphamide (monthly dosing), cyclosporin, infliximab or mycophenolate can be added to the steroid regimen (32). Major advancements include the need for a laboratory panel for glomerulonephritis to check for signs of active autoimmune-associated damage to the nephron (ANA or anti-nuclear antibody, rheumatoid-factor antibody, ANCA or anti-neutrophil cytoplasmic antibody, dsDNA or double-stranded DNA; C3/C4 or complement protein 3/4 levels, CH50 or total complement levels, SPEP/UPEP or serum/urine protein electrophoresis and hepatitis panel).

### Neurologic

Neurologic irAEs are rare but can have significant impacts on patient quality of life. The management for specific subtypes mostly include infection rule-out, CNS imaging/PNS stimulation testing and IO temporary hold (mild/moderate) or permanent discontinuation. Certain rare reactions such as Guillain-Barre syndrome (GBS), myasthenia gravis (MG) and transverse myelitis are managed with the current standard of care and neurology consultation as well as inpatient or ICU admission (26, 32).

#### Aseptic Meningitis/Encephalitis

Meningitis/encephalitis irAEs require MRI brain with/without contrast (pituitary protocol included), lumbar punctures and neurologic consultation (26, 32). For a more encephalitic picture, patients should also receive electroencephalogram (EEG), inflammatory/general laboratory biomarkers, vasculitis panel, thyroid panel and paraneoplastic panel of both CSF and serum (35). For mild irAEs IO is held and discontinued for severe cases. Severe cases also require inpatient care with a thorough bacterial/viral infection rule out, as well as possible empiric treatment with IV acyclovir (35). If infection is ruled out, close monitoring without steroids can be performed or steroids can be given with prednisone 0.5-1 mg/kg/day (mild) or methylprednisolone 1-2mg/kg/day (moderate-severe) (26, 32). For encephalitis, IVIG or plasmapheresis can be added to the steroid regimen for 3-5 days if oligoclonal bands are present on LP or if symptoms progress (35). If no improvement is observed within 7-14 days of steroids or autoimmune encephalitis antibody is found, rituximab can be added to the regimen (32). Advancements include no longer obtaining morning cortisol levels to test for central adrenal insufficiency for patients with purely CNS irAEs due to the insignificant impact on overall management (32).

#### Peripheral Neuropathy

Neuropathy is the most common irAE of the neurologic subtype and is also a common adverse effect of many oncologic treatments, which is an important consideration. Grade of neuropathy are based on the severity and degree of peripheral nerve involvement (32). Grade 1 reactions should have thorough medication reconciliation, infection and autoimmune work-up, metabolic/endocrine screening and chemical exposure/trauma history (35). For grade 1, IO is held and symptoms are monitored for a week (32). For grade 2, an additional neurologic consultation, neuraxial imaging, electromyography and/or nerve conduction studies are recommended (32). Grade 2 reactions are managed by holding IO and close monitoring or steroid therapy dosed at 0.5-1 mg/kg/day. If progression is observed, methylprednisolone 2-4 mg/kg/day is initiated (32). For grades 3-4 the management follows the same algorithm as that of the GBS treatment (26, 32). Gabapentin, pregabalin or duloxetine can be offered if there is associated neuropathic pain (26, 32). Major advancements include following GBS protocols for severe peripheral neuropathies from irAEs.

### Ophthalmologic

Visual changes associated with ICIs are rare but should be closely monitored given the impact on patient morbidity and quality of life. Visual irAEs are split into two major categories uveitis and episcleritis (32). Patients should have an ophthalmology consultation and visual work-up: acuity and color testing, pupil size/shape/reactivity testing, red reflex, fundoscopic evaluation and possibly more invasive testing (26, 32). Mild or grade 1 uveitis and episcleritis are managed conservatively with artificial tears, IO continuation and monitoring, in which IO can be held if symptoms worsen (32). For grades 2-4, IO therapy is held and patients are provided with local ophthalmic and systemic steroids (prednisone/methylprednisolone) (26, 32). Major advancements for ocular irAEs have been the less aggressive use of ICI discontinuation in favor of treatment continuation in milder presentations due to likely oncologic benefits.

### Endocrine

#### Hyperglycemia

Hyperglycemia is a unique irAE that could be linked to an iatrogenic type I diabetes mellitus (T1DM), however, many other cancer treatment agents can cause elevated blood sugar as well. When considering an irAE as the cause of hyperglycemia, the degree of blood sugar elevation and the presence of DKA determine the work-up and management. If patients have fasting hyperglycemia < 200 g/dL and/or a history of T2DM, the elevation can be attributed to pre-existing T2DM or medication-induced hyperglycemia (most commonly steroids) (32). If the patient has a fasting glucose > 200, random > 250 or a history of T2DM with a fasting/random >250, patients should be considered to have new onset T1DM, which requires comprehensive T1DM laboratory panel (C-peptide and anti-GAD, anti-islet cell antibodies) and an evaluation for DKA (blood pH, BMP, urine/serum ketones, anion-gap, BHB) (26, 32). If DKA is negative, IO is continued, and blood glucose is managed with lifestyle modification. Endocrinology can be consulted if persistent elevations are observed. If DKA is positive, IO is held until DKA resolves and standard inpatient DKA management with endocrinology consultation is recommended (32, 36).

#### Thyroid

Thyroid irAEs are one of the more common endocrine irAEs. This is why the management of endocrine irAEs can allow for IO continuation despite adverse effects due to the availability of hormone replacement therapy (32). According to the current literature, thyroid abnormalities associated with IO are more frequent with anti-PD1 targeted than anti-CTLA-4 agents (17).

For hypothyroid states, thyroid irAEs are split based on the degree of symptoms at presentation. For asymptomatic/subclinical hypothyroid states, thyroid function tests (TFTs) which include TSH and free T4 are monitored every 4-6 weeks. The following management is based on whether TSH and free T4 adjust appropriately (26, 32). If TSH is 4-10 or elevated (>10) and T4 is normal, IO can be continued (26, 32). If TSH elevation is from 4-10 than the patients can continue with TFT observation. If TSH is >10 than levothyroxine is considered. For low-normal TSH levels and low T4, central hypothyroidism must be considered, which includes brain imaging (MRI) as well as pituitary hormone panel (ACTH, LH, FSH, TSH, testosterone/estrogen) (32). The management of this scenario can be seen in the pituitary/hypophysitis work-up below.

For symptomatic thyrotoxic states or presenting with laboratory evidence of low TSH and/or high free T4/total T3, IO can be continued with likely endocrine consultation. Patients should be managed with propranolol 10-20 mg q4-6h or atenolol/metoprolol with repeat TFTs in 4-6 weeks (32). If normal, beta-blockers can be stopped as the most frequent etiology of this associated thyrotoxicosis is painless thyroiditis, which spontaneously subsides within a couple months (37). However, if persistent, Graves’ disease evaluation should be pursued. In addition, thyrotoxicosis is followed by hypothyroid state in 50-90% of patients (32). Therefore, endocrine consultation for subsequent levothyroxine dosing is recommended.

#### Pituitary or Hypophysitis

Hypophysitis is a significant irAE that has a stronger association with CTLA-4 agents over PD-1/PD-L1 ICIs (37). Patient should receive evaluation with morning cortisol, ACTH, TSH, free T4, LH, FSH, sex hormones and an MRI brain +/- contrast with pituitary sellar cuts if symptomatic (32). Management includes endocrine consultation, IO hold and initiation of appropriate hormone replacement depending on labs observed (Secondary adrenal insufficiency: low ACTH, low cortisol; central hypothyroidism: low TSH, low free T4). If acute and severely symptomatic, patients can also have high-dose steroids provided (38).

#### Primary Adrenal Insufficiency (PAI)

PAI is an extremely rare irAE, often not associated with the ICI itself. Thus, PAI should have endocrinology consultation for further work-up of an alternative etiology to irAEs. Similar to many of the other endocrinopathies, the symptom presentation can be subtle requiring keen observation from the treating physician and a comprehensive laboratory panel depending upon the CTCAE grading. Acute hypocortisolemia, in particular, can be life threatening if missed and requires thorough rule out even when symptoms appear mild (38).

### General irAEs (Fatigue)

Fatigue is a non-specific irAE that is commonly associated with numerous cancer treatments and oncologic disease itself. This makes it particularly relevant to rule out other more insidious causes of fatigue before general irAEs can be definitively diagnosed. The work-up includes a full physical, laboratory panel (hematologic, endocrine, electrolyte) and medication review. Severity is graded from 1-4, with management for grades 1-2 (mild-moderate) mostly consisting of IO continuation (can consider hold for grade 2), close monitoring and follow-up for new symptoms (32). Other considerations include disease progression, temporary IO holds and specialty consultations (32). For grades 3-4, IO discontinuation is recommended with follow-up and possible treatment re-continuation based off the final underlying diagnosis of disease progression, irAE or alternative etiologies (32).

### Musculoskeletal MSK irAEs (Arthritis, Arthralgia, Joint Pain, etc.)

The major advancements in MSK irAEs includes the use of steroid-sparing immunomodulators for steroid-refractory scenarios, stronger recommendations for non-opioid pain regimens, specific autoantibodies to screen for and greater detail for polymyalgia rheumatica (PMR) and giant cell arteritis (GCA) management. Effectively, PMR and GCA activation or re-activation should be screened for with a history and clinically concerning symptoms, which warrant an urgent rheumatologic and ophthalmologic consultation (32). This also requires early high-dose steroids for patients with worrisome symptoms of GCA (26, 32).

#### Inflammatory Arthritis

Inflammatory arthritis associated irAEs are split into mild, moderate and severe to determine management. Diagnosis can include rheumatologic consultation if significant. Severity is based on the number of joints affected, functional assessment, imaging findings at joints, presence of auto-antibodies (ANA, anti-cyclic citrullinated peptide or anti-CCP) and the levels of inflammatory markers (CRP, ESR, rheumatoid factor) (32). Mild reactions can have IO continuation and treatment with NSAIDs or steroids 10-20 mg daily for 2-4 weeks (32). Intra-articular steroids can also be considered if NSAIDs and conventional steroids fail (26, 32). Moderate reactions can consider a hold on IO therapy and oral prednisone 0.5 mg/kg/day for 2-3 weeks. Severe reactions lead to IO discontinuation and oral prednisone/methylprednisolone 1 mg/kg/day (32). If no improvement is observed through week 1-2, urgent rheumatology consult is indicated to consider other anti-rheumatic drugs such as infliximab, methotrexate, tocilizumab, sulfasalazine, azathioprine, adalimumab, etanercept and hydroxychloroquine (32). Throughout MSK irAE management, regardless of severity, patients should have inflammatory labs (ESR, CRP) monitored every 4—6 weeks (32).

#### Myalgias (Pain) and Myositis (Weakness)

Myalgias and myositis associated with ICIs require a diagnostic work-up similar to inflammatory arthritis and the severity is divided into mild and moderate-severe. Laboratory work-up includes inflammatory markers (CMP, ESR, CRP, anti-CCP), muscle cell biomarkers (creatine kinase or CK, aldolase, troponin), strength testing, cranial nerve testing and evaluation for other concomitant irAEs (myasthenia, myocarditis) (32). Mild reactions should consider having IO therapy held, aldolase/CK monitoring and pain management with NSAIDs (32). Diagnosis of polymyalgia rheumatica/giant cell arteritis should also be considered. For moderate-severe reactions, IO should be held and treatments should include prednisone 1-2 mg/kg/day for inflammation as well as urgent consultations with rheumatology and neurology (32). Aldolase and CK should also be monitored until symptom resolution or steroids discontinuation. MRI, EMG and biopsies can be considered at effected muscle groups. If refractory to steroids, IVIG 2g/kg, plasmapheresis, infliximab, rituximab and mycophenolate can be considered as well (32).

## irAEs Areas of Need and Future Directions

While clinicians’ expertise of irAEs management has improved with the wide-use of ICI, several challenges remain. There is a need to clarify the complex network of downstream pathways associated with CTLA-4 and PD-1/PD-L1 augmentation. Specifically, this relates to their impact on irAE profiles as well as oncologic treatment outcomes (39). Additionally, irAEs display a wide range of severity and localization, some more specific than others to the cancer type being treated and the ICI class used (39). For example, immune surface receptor clustering appears to predict endocrine irAEs by ICI class. Thyroid irAEs from ICIs occur most frequently with PD-1/PD-L1 inhibitors than CTLA-4s (37). Conversely, hypophysitis occurs more often with CTLA-4s compared to PD-1/PD-L1s (37). Both of these are explained by higher rates of PD-1 and CTLA-4 receptor concentrations at the given sites respectively (37). In addition to receptors associated with ICIs, progress has been made related to assays that pinpoint specific laboratory measurements that could predict irAE occurrence. These include quantitative T cell sub-population measurements, T/B cell surface receptor concentrations, autoantibody panels, cytokine levels (IL-17 specifically) and eosinophilia. Despite this progress, larger studies are needed to confirm the practicality and efficacy of these laboratory studies prior to clinical application (40–43). Understanding these pathways can provide further scientific and clinical benefit to better our understanding of IO-related immune dysregulation.

In addition, steroid-sparing based agents can offer alternative therapeutic options for oncologic patients. This can reduce the long-term adverse effects of steroids, address steroid-resistant irAEs and provide options for patients with contraindications to steroid therapy (DM, metabolic syndrome, psychosis, etc.) (44). In the future this could allow treating clinicians to use steroid-sparing agents earlier and save high-dose steroids for symptom progression or more severe situations (44).

**Figure 3:**
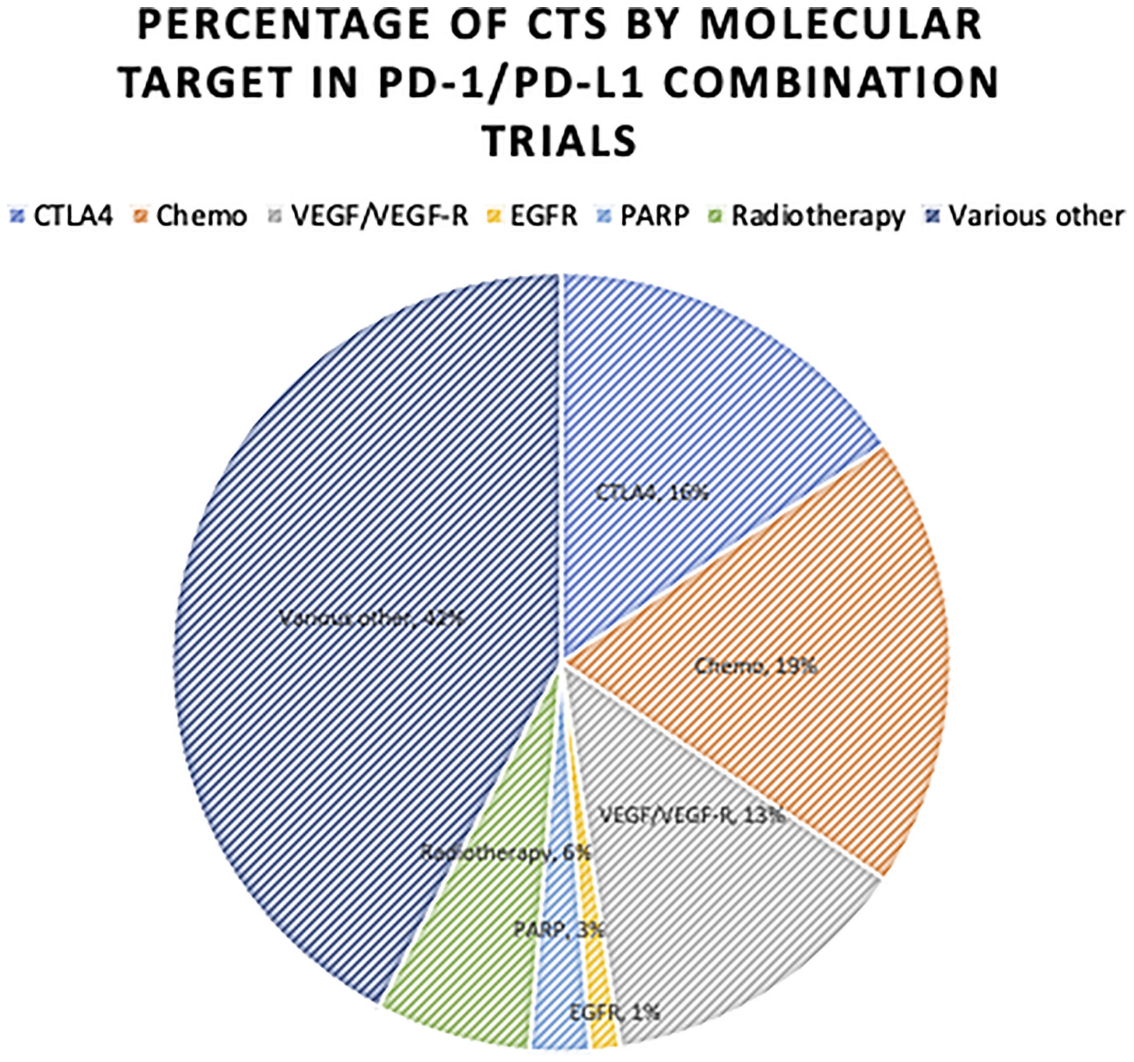
Proportion of Combination RCTs for PD-1/PD-L1 Combination Therapy by Target Studied. Proportion of current RCTs studying other targets in combination with PD-1/PD-L1 agents. IO, Immuno-Oncologic Therapy; RCT, Randomized Control Trial; ICI, Immune-checkpoint Inhibitor; CTLA-4, Cytotoxic T-Lymphocyte Antigen-4; PD-1, Programmed Death Receptor-1; PD-L1, Programmed Death Receptor Ligand-1; VEGF/VEGF-R, Vascular Endothelial Growth Factor/-Receptor; EGFR, Epidermal Growth Factor Receptor; Chemo, Chemotherapy; PARP, Poly ADP Ribose Polymerase. Various Other, Includes a large array of additional targets. Only significant combination targets were included.

**Figure 4:**
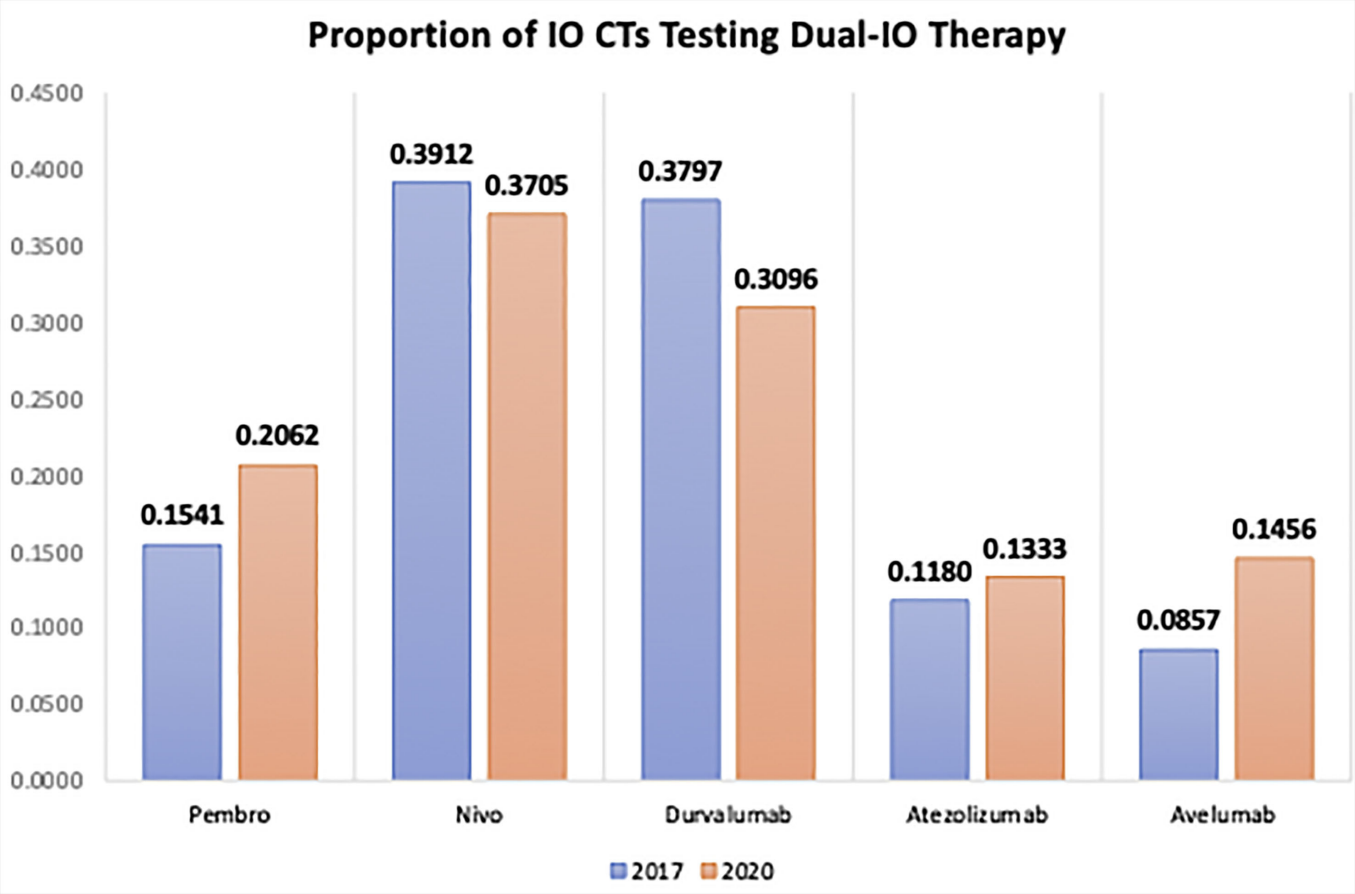
Percentage of ICI Agents Studied in Combination as a Proportion of the Total Trials Per Agent. The percentage of current RCTs that are studying the following ICI agents in combination strategies organized by ICI class. IO, Immuno-Oncologic Therapy; RCT, Randomized Control Trial; ICI, Immune-checkpoint Inhibitor; Pembro, pembrolizumab; Nivo, nivolumab.

With the increasing use of combination regimens, the rates and severity of irAEs are expected to rise compared to ICI monotherapy. This is evident in the rates of irAEs by ICI regimen as seen in **Figure 2**. Additionally, combination regimens may cause synergistic irAE activation through more complex mechanisms relative to monotherapy. **Figures 3** and **4** show just how significant a role combination regimens will play in the future, given the large proportion of current IO clinical trials under study (2). **Figure 3** (2) findings are based on the major cellular targets that are being studied in combination with PD-1/PD-L1 agents as a percentage of total PD-1/PD-L1 combination trials. **Figure 4** (2) shows the percentage of total IO trials that are testing combination therapies currently. Further, in IO-based combinations therapy including chemotherapy or targeted therapy agents, it might be difficult to discern whether a symptom such as diarrhea is irAEs or a side effect of the non-IO drug(s) included in the combination.

Clinical trials that lead to ICI therapy approval frequently excluded patients with pre-existing autoimmune diseases. However, there are nine studies that have monitored these patient populations with pre-existing immunologic pathologies. Polymyalgia rheumatica, myasthenia, rheumatoid arthritis and psoriasis/psoriatic arthritis all appeared to display the highest rates (>50% of patients receiving ICIs) of autoimmune reactivation/flares with ICI therapy (5). For irAE prevention the main goal is to risk stratify patients prior to treatment. A wide range of research is studying associated laboratory/clinical findings that can allow better identification of high-risk patients and the most common irAEs by malignancy and ICI class (17, 45, 46).

Studies examining the potential for pre-ICI treatments, in which steroids were given before ICI initiation, showed little to no efficacy with regards to the rates of irAEs (47). In steroid-resistant irAEs, anti-TNF-alpha agents have displayed efficacy in controlling uveitis, colitis and hepatitis (48). Additional studies have found efficacy for steroid-resistant irAEs with cyclophosphamide and mycophenolate for pneumonitis; and methotrexate and hydroxychloroquine for arthritis (30). The role of other immunomodulators in irAE management can be seen in **Table 3**.

While some irAEs are associated solely with a negative side effect of ICIs, there is research showing positive correlations between certain irAEs and oncologic outcomes. Certain dermatologic irAEs, such as vitiligo, may be positive prognostic indicators for melanoma patients (49). Similar associations with efficacy were also found with thyroid cancer, renal cell carcinoma, among other cancers irAEs (50–52). Additionally, adverse events associated with non-IO therapies, such as Sunitinib, show similar associations and impacts to baseline thyroid function in patients with metastatic renal cell carcinoma (53). These irAEs may require further study of their management to allow for optimal ICI continuation due to the associated impact on cancer treatment.

Oncologic-specific algorithms may also be necessary for certain aggressive tumors that have complex interactions with the endocrine, humoral, and cellular-based immune systems. Studies examining the impact of IO agents on certain high-grade neuroendocrine (HG-NEN) tumors have shown mixed results raising questions related to the predictive/prognostic value of PD-1/PD-L1 expression alone for IO deployment and side-effect management (54). While some NENs such as melanoma and non-small cell lung cancer (NSCLC) showed promising results regarding PD-1/PD-L1 expression and ICIs, the management recommendations for certain cancers, such as merkel cell carcinoma (MCC), requires additional clinical trial data to better inform IO management (55).

Current controversies within the irAE literature exist mostly in how irAEs are reported, documented and, therefore, managed. Outside of endocrine irAEs, which have specific laboratory cutoffs and have limited alternative diagnosis, there is significant inconsistency between providers and institutions for irAE reporting (31). Thus, there is a significant need for standardization of terminology, documentation and diagnostic parameters within the irAE research space (56).

IrAEs challenge clinicians to weigh the costs and benefits of these new IO therapies. IrAEs are complex and display associations and disassociations with oncologic outcomes. Currently, irAE management algorithms, documentation patterns and pre-ICI screenings appear to be at the forefront of irAE research and could revolutionize the way we manage these side effects.

## Conclusion

As the use of IO therapy continues to expand to combination regimens and to the adjuvant/neoadjuvant treatment spaces, it becomes an ever-pressing issue that our understanding of irAEs improves and that irAE management evolves. Thus far, significant strides have been made to identify high-risk patients, high-risk irAEs and construct a treatment arsenal consisting mostly of steroids and immunomodulators (anti-TNF, rituximab, mycophenolate, etc). With time, this field will continue to expand and allow for further optimization of IO therapy for patients. This can lead to reduced costs, improved oncologic outcomes and maximized quality of life for patients dealing with cancer and IO therapy.

## Author Contributions

TO primarily wrote the manuscript and executed suggested revisions provided by the included authors. BN provided supervision and advisory during the writing and submission process. DM also assisted in revisions and manuscript submission. All authors contributed to the article and approved the submitted version.

## Conflict of Interest

MB has acted as a paid consultant for and/or as a member of the advisory boards of Exelixis, Bayer, BMS, Eisai, Pfizer, AstraZeneca, Janssen, Genomic Health, Nektar, and Sanofi and has received grants to his institution from Xencor, Bayer, Bristol-Myers Squibb, Genentech/Roche, Seattle Genetics, Incyte, Nektar, AstraZeneca, Tricon Pharmaceuticals, Peleton Therapeutics, and Pfizer for work performed as outside of the current study. BN has acted as a paid member of the advisory board of Exelixis.

The remaining authors declare that the research was conducted in the absence of any commercial or financial relationships that could be construed as a potential conflict of interest.

## Publisher’s Note

All claims expressed in this article are solely those of the authors and do not necessarily represent those of their affiliated organizations, or those of the publisher, the editors and the reviewers. Any product that may be evaluated in this article, or claim that may be made by its manufacturer, is not guaranteed or endorsed by the publisher.
